# Crystal phase engineering of self-catalyzed GaAs nanowires using a RHEED diagram†

**DOI:** 10.1039/d0na00273a

**Published:** 2020-04-13

**Authors:** T. Dursap, M. Vettori, A. Danescu, C. Botella, P. Regreny, G. Patriarche, M. Gendry, J. Penuelas

**Affiliations:** Institut des Nanotechnologies de Lyon-INL, UMR 5270 CNRS, Université de Lyon, École Centrale de Lyon 36 avenue Guy de Collongue F-69134 Ecully cedex France jose.penuelas@ec-lyon.fr; Université Paris-Saclay, CNRS, Centre de Nanosciences et de Nanotechnologies 91120 Palaiseau France

## Abstract

It is well known that the crystalline structure of the III–V nanowires (NWs) is mainly controlled by the wetting contact angle of the catalyst droplet which can be tuned by the III and V flux. In this work we present a method to control the wurtzite (WZ) or zinc-blende (ZB) structure in self-catalyzed GaAs NWs grown by molecular beam epitaxy, using *in situ* reflection high energy electron diffraction (RHEED) diagram analysis. Since the diffraction patterns of the ZB and WZ structures differ according to the azimuth [11̄0], it is possible to follow the evolution of the intensity of specific ZB and WZ diffraction spots during NW growth as a function of the growth parameters such as the Ga flux. By analyzing the evolution of the WZ and ZB spot intensities during NW growth with specific changes of the Ga flux, it is then possible to control the crystal structure of the NWs. ZB GaAs NWs with a controlled WZ segment have thus been realized. Using a semi-empirical model for the NW growth and our *in situ* RHEED measurements, the critical wetting angle of the Ga catalyst droplet for the structural transition is deduced.

## Abstract 2

It is well known that the crystalline structure of the III–V nanowires (NWs) is mainly controlled by the wetting contact angle of the catalyst droplet which can be tuned by the III and V flux.

## Graphical abstract

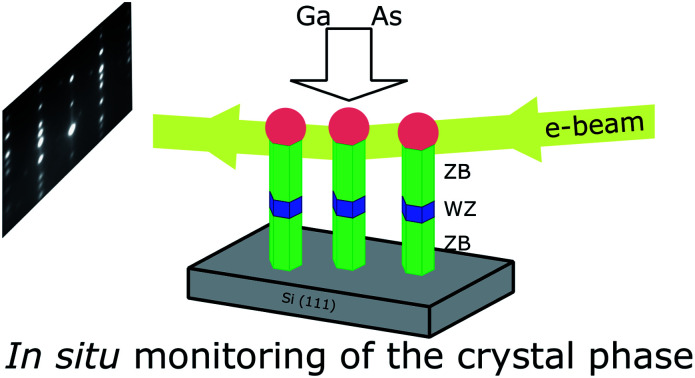

## 1. Introduction

Semiconductor nanowires (NWs) are greatly promising materials for future nanoelectronic and nanophotonic devices.^1–5^ The fabrication of these NWs is mainly based on the vapor–liquid–solid (VLS) growth mechanism, a method where atoms are transported from the vapor phase to the NW solid phase through a liquid catalyst droplet.^6^ The occurrence of two crystal phases in non-nitride III–V NWs has attracted considerable attention in the last decade since the model developed by F. Glas *et al.* in 2007 to explain the nucleation of the Wurtzite (WZ) or the Zinc Blende (ZB) crystal phase in such NWs.^7^ Indeed, while only the ZB phase is reported in bulk III–V materials, III–V NWs exhibit the ZB or WZ phase depending on the growth parameters. It has been theoretically predicted that WZ GaAs has a slightly larger band gap than its ZB counterpart, with a positive conduction-band offset of up to 149 meV (ref. 8) and a slightly different band structure. Moreover, ZB and WZ phases are known to exhibit different electronic,^9^ optical,^10–12^ mechanical,^13^ thermoelectric^14^ and piezoelectric properties.^15,16^ These tunable physical properties only arising from the crystalline structure without incorporation of foreign chemical elements are particularly attractive for device fabrication. The possibility of controlling the crystal phase of GaAs NWs during growth opens the possibility of developing a wide range of heterostructured NWs including quantum dots, quantum disks as thin as a single monolayer (ML) and superlattices.^17^

The WZ phase is often obtained for gold-catalyzed III–V NWs, while the ZB phase is mostly obtained for self-catalyzed ones. In the latter case, the WZ phase is, however, often observed at the NW top near the Ga droplet and can be ascribed to the end of NW growth when the Ga flux is stopped and the Ga droplet is consumed under the As flux.^18–24^ The control of the ZB and WZ phases in self-catalyzed GaAs NWs has thus become a major challenge. Based on the models of F. Glas^7^ and V. Dubrovskii,^25^ occurrence of the WZ or ZB phase in self-catalyzed GaAs NWs has been explained by the position of the nucleation for a new atomic layer either at the triple phase line (TPL) or inside the droplet, respectively.^19,22,26–28^ It was recently shown that the nucleation position mainly depends on the droplet wetting angle and therefore on the catalyst droplet volume, for both Au-catalyzed and self-catalyzed GaAs NWs.^29–32^ A critical wetting angle *β*_c1_ in the 121°–124° range for Au catalyzed NWs^29,30^ and of about 125°–127° for self-catalyzed NWs^31,32^ has been experimentally observed for a transition from the WZ to the ZB crystal phase above this critical angle. In addition, a second critical angle *β*_c2_ in the 85°–100° range below which a transition from the WZ to the ZB crystal phase is also expected to occur has been experimentally observed.^32^ From the *in situ* transmission electron microscopy (TEM) observations by Jacobsson *et al.*, above this angle a truncated facet is present at the NW top facet, thus determining that the nucleation site is inside the droplet leading to the ZB crystal phase.^29^ In self-catalyzed growth, the Ga droplet volume is mainly dependent on the growth conditions, in particular on the Ga and As fluxes. Many previous studies have thus reported the control of the GaAs NW crystal phase by tuning the Ga and/or As fluxes.^22,27,31,33–37^ In these studies the crystal phase characterization has been mainly performed *ex situ* by TEM. However, an *in situ* and real-time characterization tool also appeared to be very useful in order to characterize and possibly tune the crystal phase of the self-catalyzed GaAs NWs during growth. Compared to *in situ* X-ray diffraction^35^ and *in situ* TEM,^29,30^ the reflection high energy electron diffraction (RHEED) technique is commonly coupled with molecular beam epitaxy (MBE) to follow the structural properties of growing layers and nanostructures. Despite this, there are relatively few studies reporting RHEED observations during the self-catalyzed GaAs NW growth. Scarpellini *et al.*^24^ reported on the consumption of the Ga droplets at the end of the growth, while Rudolph *et al.*^21^ reported on the influence of the As flux on the InAs NW structural properties and Bastiman *et al.* reported on the incubation time of GaAs NWs.^38^ Only recently, *in situ* RHEED characterization of the NW growth coupled with *ex situ* TEM measurements was reported by Jo *et al.*^39^

In this work, we focus on the characterization of the growth of self-catalyzed GaAs NWs on a Si(111) substrate using *in situ* RHEED. In particular, we aim to control the formation of the ZB or WZ crystal phase of the GaAs NWs as a function of the Ga flux amounts by using the RHEED pattern. TEM measurements of some NWs were performed to check the obtained crystal phases.

## 2. Experimental

All the samples were grown on epi-ready Si(111) substrates using a solid-source MBE reactor. The native oxide on the substrates was preserved to enable self-catalyzed growth.^40^ Each substrate was only cleaned in acetone and ethanol solutions for 10 min. The substrates were degassed at 200 °C in an ultra-high vacuum and introduced inside an MBE reactor. One ML of Ga was pre-deposited at 480 °C to form Ga droplets.^41,42^ The sample temperature was then increased to the NW growth temperature *T*_G_ = 600 °C and the growth was initiated by the simultaneous opening of the Ga and As fluxes. The MBE system was supervised using homemade software which allows fine control of the Ga and As_4_ fluxes, shutters and valves. Under standard conditions, the NWs were grown with Ga and As_4_ fluxes equal to 0.5 ML s^−1^ and 1.15 ML s^−1^, respectively, quoted in units of equivalent growth rates of GaAs 2D layers measured by RHEED oscillations on a GaAs substrate,^21^ thus corresponding to a V/III flux ratio = 2.3. RHEED measurements were systematically performed with the electron beam energy set to 30 keV to obtain information on the crystal structure of the NWs during the growth process. The sample rotation was systematically stopped during the growth to record the RHEED patterns and no significant difference was observed in the NW morphology and crystal structure compared to those of the NWs grown with continuous rotation. The samples were then observed with a JEOL scanning electron microscope (SEM) using an acceleration voltage of 10 kV and TEM measurements were performed on a FEI Titan Themis 200 working at 200 kV.

## 3. Results

### 3.1. GaAs NW crystal structure

**Fig. 1:**
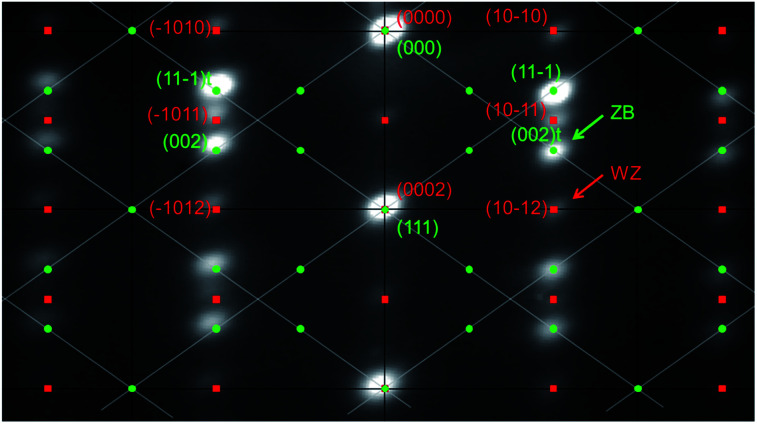
RHEED pattern recorded along the [11̄0]azimuth, where ZB and WZ spots are visible, superposed with an indexation diagram. Green dots represent the ZB plans taking into account the two ZB variants. Red squares represent WZ plans. The ZB and WZ spots whose intensities are measured are indicated by green and red arrows, respectively. An extinction of the structure factor *F*_*hkl*_ is responsible for the missing spots of one over two ZB columns and of the one over two WZ spots along the growth axis. The spots visible between the bright spots along the growth axis are present due to other orders of diffraction.

**Fig. 2:**
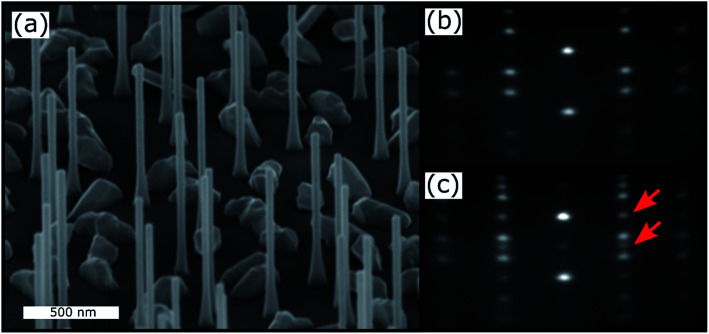
(a) SEM image (45° tilted) of the measured sample. (b) RHEED pattern recorded along the [11̄0] azimuth during the NW growth, where only ZB spots are visible. (c) RHEED pattern recorded at the end of the NW growth after closing the Ga flux, where WZ spots are also visible (indicated by red arrows).

**Fig. 3:**
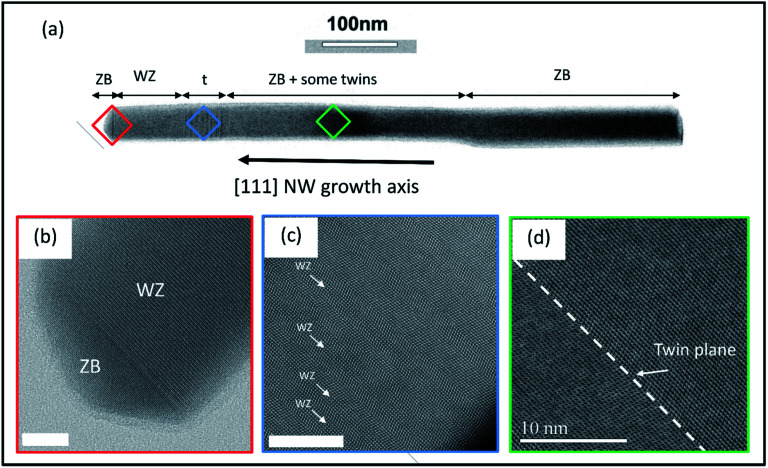
(a) (HR)-STEM image (with the [1–10] zone axis) of a typical GaAs NW where different zones can be identified: (b) the pure-WZ segment and the final ZB segment grown at the end of the droplet consumption, (c) the transition zone t with a high density of twin planes and ZB/WZ phase mixing, (d) the pure-ZB phase of the NW with some twin planes for a NW length greater than ∼200 nm. Scale bars in (b), (c) and (d) are 10 nm. (a), (b) and (d) show BF-STEM images while (c) shows a HAADF-STEM image.

A typical RHEED pattern measured along the [11̄0] azimuth of self-catalyzed GaAs NWs when both the WZ and ZB phases are present is shown in Fig. 1. The position of the spots is in agreement with epitaxial growth of NWs on Si(111): the GaAs [111] and [11̄0]axes are parallel to the Si [111] and [11̄0] axes, respectively. The corresponding spots of the WZ phase and twinned ZB phase are indexed. The (10–12) WZ and (002)*t* ZB spots whose intensities will be measured during growth are indicated by the green and red arrows, respectively. The SEM picture in Fig. 2(a) shows the NW morphology after 10 min of growth: their length is about 1.1 μm and their diameter is in the 40–60 nm range, with a density close to 1 NW μm^−2^. Some parasitic nanocrystals among the NWs are evidenced; however the substrate surface is still visible since the growth temperature was optimized in order to minimize this parasitic growth. Fig. 2(b) shows the typical RHEED pattern obtained along the [11̄0]azimuth during the NW growth. Only ZB spots are observed indicating the growth of pure ZB NWs with twin planes. Fig. 2(c) shows the RHEED pattern at the end of the NW growth after closing the Ga shutter and cooling the sample under As_4_ flux. We can observe the presence of low intensity WZ spots (two of them are indicated by red arrows). Fig. 3(a–d) show TEM and HRTEM images (with the [1–10] zone axis) of a typical self-catalyzed GaAs NW. Under the applied growth conditions (*T*_G_ = 600 °C and V/III flux ratio = 2.3) the NW exhibits a pure-ZB phase almost over its entire length. Both ZB variants can be observed due to the presence of some twin planes (marked in green in Fig. 3(a) and shown in Fig. 3(d)). Then, near the NW top, we observe a sequence with a transition zone about 60 nm in length with a high density of twin planes and some WZ segments (marked in blue in Fig. 3(a) and shown in Fig. 3(c)), and finally a pure WZ segment about 100 nm in length followed by a thin ZB segment about 10 nm in length (marked in red in Fig. 3(a) and shown in Fig. 3(b)).

**Fig. 4:**
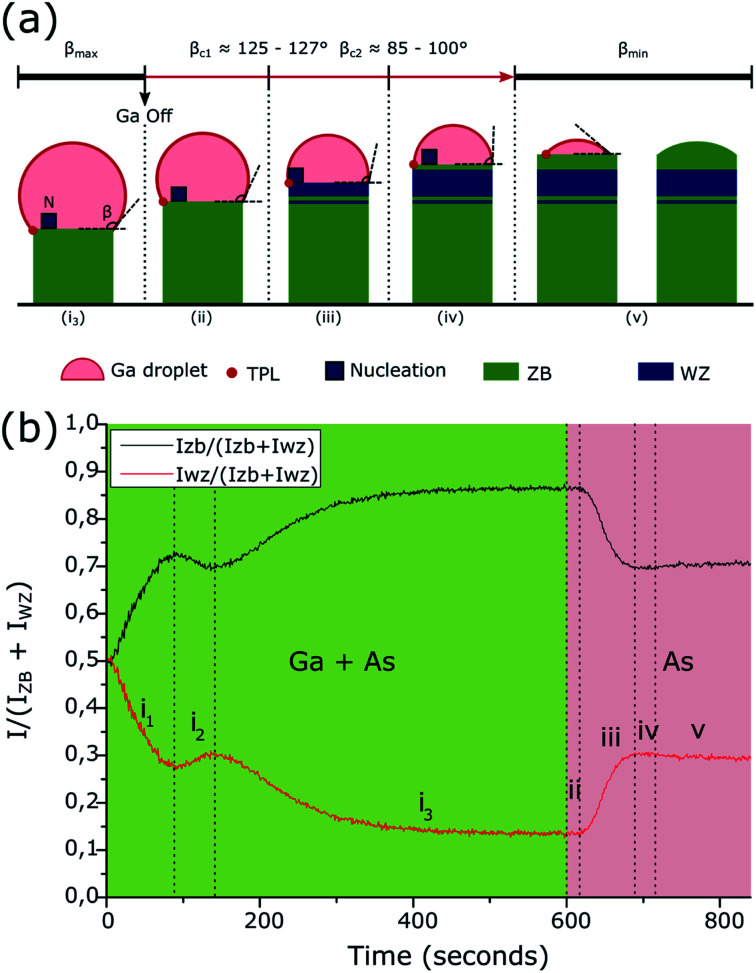
(a) Scheme of Ga droplet evolution: (i_3_) during the growth of the GaAs NWs with Ga and As flux leading to a wetting angle *β* = *β*_max_ larger than *β*_c1_ = 125°–127°, with nucleation inside the droplet thus to a ZB phase. Then, after closing the Ga flux, the droplet volume and wetting angle started to decrease leading to (ii) nucleation inside the droplet and thus to a ZB phase as long as the wetting angle is larger than *β*_c1_ = 125°–127°, (iii) nucleation at the TPL when *β*_c1_ = 125°–127° < *β* < *β*_c2_ = 85°–100° and thus to a transition zone t with ZB/WZ phase mixing followed by a pure-WZ segment, (iv) nucleation inside the droplet and thus to a short ZB segment when the wetting angle becomes lower than *β*c_2_ ≈ 85°–100°, until (v) the wetting angle reaches *β*_min_, hence ending the growth with the final consumption of the droplet. (b) 
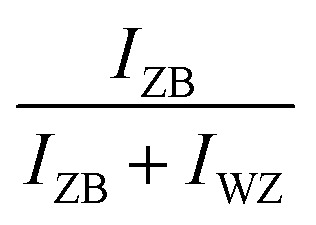
 and 
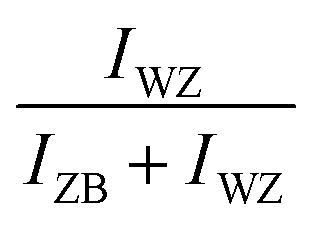
 intensity ratios as a function of the growth time. The growth under Ga and As flux is shown in green and the growth under As flux only is shown in red.

This final sequence and the associated growth mechanism are well known thanks to previous studies.^18–24,26^ Indeed, the GaAs NW growth continues with the consumption of the Ga droplets leading to a decrease of its volume. Recent *in situ* TEM results obtained on Au- or Ga-catalyzed GaAs NWs^29–32^ have confirmed that nucleation of the ZB or WZ structure will occur depending on the droplet shape and more precisely on the wetting angle *β* which determines the location of the atomic layer nucleation. From these recent results, and as illustrated in Fig. 4(a), the final structural sequence of the NWs can be explained as follows depending on *β*: first, the NW growth is presumed to be with a large wetting angle *β*_max_,^31^ typically greater than *β*_c1_ = 125°–127°, leading to nucleation inside the droplet, thereby giving the growth of a ZB phase according to the F. Glas model^7^ (Fig. 3(d) and 4(a) stage (i_3_)). Once the Ga flux is closed, the droplet starts to be consumed leading to a decrease of its volume and so of the wetting angle *β*, hence leading to nucleation at the TPL thereby giving the growth of a WZ phase, also according to the F. Glas model.^7^ This step leads first to the same ZB phase, as long as *β* is greater than *β*_c1_ (Fig. 4(a) stage (ii)), and then to a ZB/WZ phase mixing segment followed by a pure-WZ segment, when *β* becomes smaller than *β*_c1_ (Fig. 3(c and b) and Fig. 4(a) stage (iii)). Once *β* becomes typically lower than *β*_c2_ = 85°–100° a final ZB segment is formed (Fig. 3(b) and 4(a) stage (iv)) up to *β*_min_ where the droplet is unpinned from the top edge of the NW and moves freely on the (111) top facet maintaining a wetting angle equal to *β*_min_ and thus also leading to a ZB phase, according to previous studies,^24,26^ until total consumption of the droplet (Fig. 4(a) stage (v)).

The first purpose of this work was to follow this structural evolution *via* the RHEED pattern during NW growth and at the growth end when the Ga flux is closed. To proceed, RHEED videos were recorded along the [11̄0] azimuth during the NW growth and the time-dependent intensities of the ZB and WZ spots were analyzed. The analyzed spots giving the ZB and WZ phase “intensities” are indicated in Fig. 1. Fig. 4(b) shows the evolution of the 
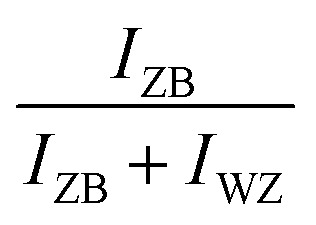
 (or 
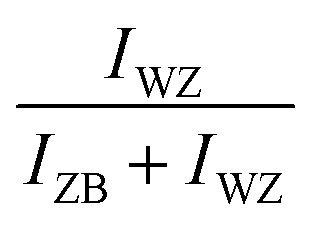
) intensity ratio (IR), where *I*_ZB_ and *I*_WZ_ are the intensities of the ZB and WZ spots, respectively, as a function of the growth time. The green area corresponds to the NW growth with Ga and As fluxes (zones (i)). At time *t* = 0 seconds, WZ and ZB IRs are equal to 0.5, due to the absence of NWs (the measured intensities are those measured on the diffraction line of the Si(111) substrate surface). Once the Ga and As fluxes are opened, an increase of the ZB IR 
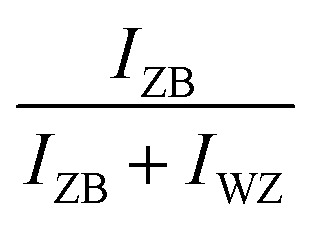
 (correspondingly, a decrease of the WZ IR 
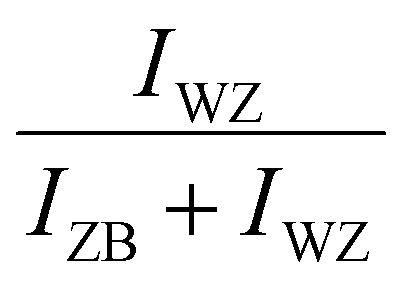
) is observed due to the growth of the parasitic ZB GaAs nanocrystals during the first few seconds, and then due to the nucleation and the growth of the NWs after a few tens of seconds^38^ (zone (i_1_)). After 90 seconds, we observe a short and slight decrease of the ZB IR (correspondingly, a short and slight increase of the WZ IR) for about 40 seconds (zone (i_2_)). Subsequently, we again notice an increase of the ZB IR (correspondingly, a decrease of the WZ IR), which tends to become constant with the growth time with a value close to 1 for the ZB IR (correspodingly close to 0 for the WZ IR) (zone (i_3_) and the corresponding stage (i_3_) in Fig. 4(a)), meaning that the NWs are entirely or quasi-entirely ZB at this moment (see Fig. 3(a)).

After 600 seconds, when the Ga flux is closed, we can assume that the Ga droplet volume starts to decrease thus leading to the sequence schematized in stages (ii) to (v) of Fig. 4(a). A delay is observable (zone (ii) in Fig. 4(b)) between the closing of the Ga flux and the beginning of the WZ IR increase (correspondingly, the ZB IR decrease). This delay measured at around 20 seconds is interpreted to be due to the consumption time of the droplets which is necessary to reach the wetting angle *β*_c1_ = 125°–127° leading to the WZ phase (corresponding to stage (ii) in Fig. 4(a)). Then, we observed an increase of the WZ IR during about 70 seconds (zone (iii) in Fig. 4(b) corresponding to stage (iii) in Fig. 4(a)), associated with the growth of the ZB/WZ phase mixing segment (zone *t* in Fig. 3(c)) and then with the pure-WZ segment (see Fig. 3(b)). A weak decrease of the WZ IR (zone (iv) in Fig. 4(b) corresponding to stage (iv) in Fig. 4(a)) is then visible and associated with the growth of the final ZB segment (see Fig. 3(b)) when *β* becomes typically lower than *β*_c2_ = 85–100°. It can be noted that this WZ-to-ZB phase transition is abrupt, not giving rise to a zone with structural defects. Finally, a stabilization of both ZB and WZ IRs is observed (zone (v) in Fig. 4(b) corresponding to stage (v) in Fig. 4(a)) and is associated with the end of NW growth when *β* = *β*_min_, with the unpinning of the droplets from the top edge of the NWs^24,26^ and the final consumption of the Ga droplets. These RHEED measurements are perfectly in line with the structural evolution of the NWs observed by TEM measurements and we can therefore associate them with the size and wetting angle modifications of the Ga droplets, as illustrated in Fig. 4(a).

It should be noted that the observed RHEED diagram can be affected by the density and the length of the NWs, as well as by the incidence angle of the electron beam due to shadowing effects. All of these parameters can modify the RHEED probed depth. Nevertheless, in our specific configuration we estimated this probed depth to be around 400–500 nm. This estimation was obtained as explained in the ESI (Fig. 1 S.I.†). However, the purpose of this work is to monitor the evolution of the RHEED diagram during the growth, and in particular to relate the time dependence of the RHEED spots to the growing crystal phase. These shadowing effects have hence only a slight influence on our data.

### 3.2. Zinc Blende/Wurtzite alternation

**Fig. 5:**
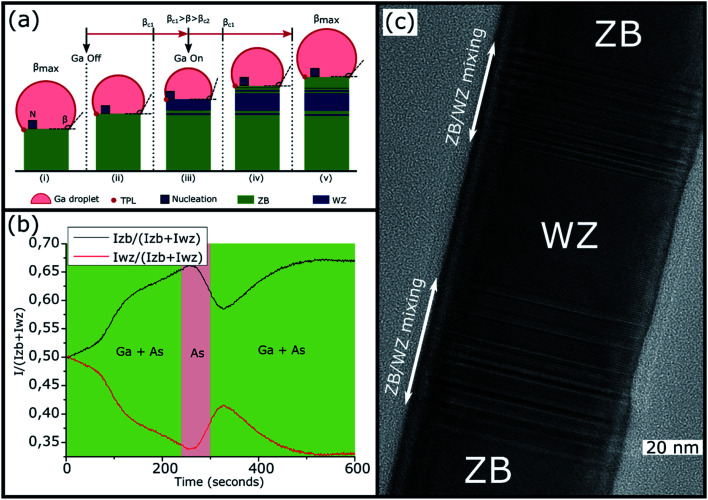
(a) Scheme of the expected Ga droplet evolution and corresponding crystal phases during the adopted procedure: (i) growth of GaAs NWs under Ga and As flux with a droplet with a wetting angle *β* = *β*_max_ larger than *β*_c1_ = 125°–127°, leading to nucleation inside the droplet and thus to a ZB phase. Then, after closing the Ga flux, the droplet volume and wetting angle started to decrease leading to (ii) a ZB phase as long as the wetting angle is larger than *β*_c1_ = 125°–127° and (iii) nucleation at the TPL and thus to a transition zone with ZB/WZ phase mixing followed by a pure-WZ segment. Then, after opening the Ga flux leading to the re-feeding of the droplet and to the increase of the wetting angle up to *β*_c1_ = 125°–127° and thus to a second transition zone with ZB/WZ phase mixing followed by a pure-ZB segment (iv). Finally, the wetting angle increases up to *β*_max_ with which the growth continues (v). (b) Intensity ratios 
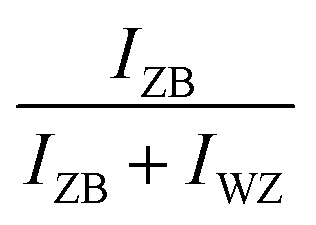
 and 
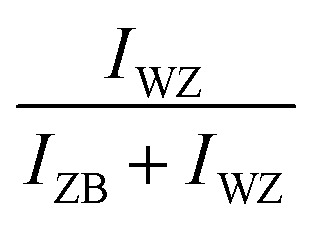
 as a function of the growth time. The growth under Ga and As flux is shown in green and the growth under As flux only is shown in red. (c) HRTEM image (with the [1–10] zone axis) showing the produced WZ segment inside a ZB GaAs NW.

**Fig. 6:**
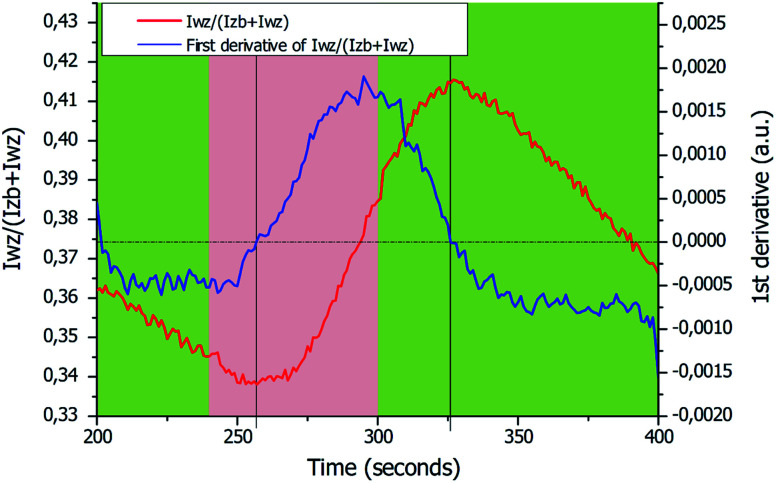
Enlarged image of the WZ intensity ratio 
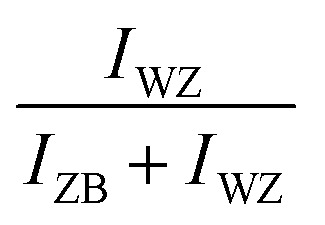
 (red curve and left axis) corresponding to the WZ segment growth. Green areas correspond to the growth under Ga and As flux and the red area corresponds to the growth under As flux only. The blue curve and the right axis are related to the first derivative of the WZ intensity ratio. The dotted black line corresponds to the zero of this curve. The times for the ZB-to-WZ and WZ-to-ZB phase transitions were extracted from the intersection between the dotted black line and the first derivative curve.

The ability to analyze the RHEED pattern and the crystal structure evolution of the GaAs NWs during growth was used to control the formation of a WZ segment inside ZB NWs. The Ga flux was stopped to reduce the size and wetting angle of the Ga droplets during a short time in order to induce the growth of a WZ segment without total consumption of the Ga droplets. For such a purpose and based on the previous results, after 240 seconds of ZB NW growth, the Ga flux was closed only for 60 seconds in order to avoid the total consumption of the droplets as analyzed from Fig. 4(b). An illustration of the expected behavior of the Ga droplets is given in Fig. 5(a), while the evolutions of ZB and WZ IRs as a function of the growth time are plotted in Fig. 5(b). At the beginning of growth the NWs exhibit a ZB structure (first green area). Then, after the Ga flux closing at 240 seconds for 60 seconds (red area), the growth of the WZ segment (including the ZB/WZ phase mixing segment) is well observable after 17 seconds with the increase of the 
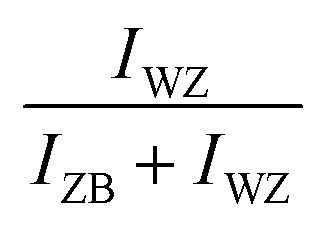
 intensity ratio. After the next Ga flux opening, it can be observed that the increase of the WZ IR did not stop immediately. This delay of about 26 seconds (see after) corresponds to Ga droplet refeeding which had not yet reached the wetting angle necessary for the transition to the ZB phase. In order to determine these durations more precisely, the first derivative of the WZ IR is plotted for the growth between 200 and 400 seconds (Fig. 6). The period without the Ga flux is represented by the red area. The ZB-to-WZ and WZ-to-ZB phase transitions are indicated by the vertical black lines, where the first derivative of the WZ IR is equal to zero. These phase transitions are used to estimate the lengths of the bottom ZB segment and of the WZ segment (including the ZB/WZ and WZ/ZB phase mixing segments) (see Table 1). In order to calculate the lengths of the bottom ZB and WZ segments, an average axial growth rate *ν* = 1.7 nm s^−1^ is used (from the average length of the bottom ZB + WZ segments measured on around twenty NWs). From Fig. 6, we approximate the growth duration of the WZ segment (including the ZB/WZ mixed segments) to be *t*_WZ_ = 69 seconds, thus corresponding to a WZ segment length of ∼117 nm. A comparison between the calculated bottom ZB and WZ segment lengths and the measured bottom ZB and WZ segment lengths obtained by TEM measurements on around twenty NWs (a typical WZ segment is shown in Fig. 5(c)) is reported in Table 1.

**Table 1:** Reported values of the growth times, the calculated lengths obtained using RHEED measurements (with an average growth rate of 1.7 nm s^−1^), the measured lengths extracted from TEM analysis and the simulated lengths. The growth times were obtained from Fig. 6. The measured lengths are average values obtained by measurements on TEM images of around twenty NWs. The interface between the ZB and WZ segments is defined from the first derivative curve of the WZ IR from RHEED analysis. The WZ segment measured by TEM includes the two ZB/WZ phase mixing segments

	Growth time (seconds)	Calculated length (nm) (from RHEED)	Measured length (nm) (from TEM)	Simulated length (nm)
Bottom ZB segment	257	∼437	440 ± 70	450
WZ segment (including the two ZB/WZ phase mixing segments)	69	∼117	120 ± 20	106

**Fig. 7:**
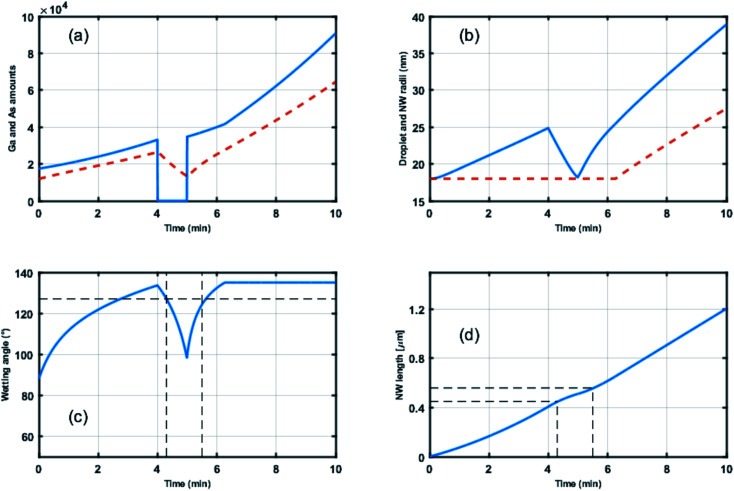
Time evolution for (a) the number of Ga atoms (continuous blue line) and As atoms (dashed red line) feeding the droplet, (b) the droplet (continuous blue line) and NW (dashed red line) radii, (c) the wetting angle and (d) the NW length. The numerical results were obtained using the following numerical values: the Ga and As sources located at incidence angles equal to 27.9° and 41°, respectively, with respect to the normal to the substrate, the nominal Ga and As fluxes given by *F*_Ga_ = 3.53 atoms/sec.nm^2^ and *F*_As_ = 27.3 atoms per sec per nm^2^. The best fit was obtained using 38 nm and 1400 nm for the diffusion lengths on the SiO_2_-terminated Si substrate and on the NW facets, respectively, and the As concentration threshold in the droplet is fixed at 1%. From the experimental data, typical values for the initial conditions for the droplet are *r* = 18 nm and wetting angle = 90°.

By extending the semi-empirical growth model for Au-catalyzed GaAs^25,33,43^ and InAs^44^ NWs, we proposed in an earlier report by Vettori *et al.*^45^ a model for self-catalyzed GaAs NWs able to account for the Ga droplet evolution as a function of (a) the Ga and As atoms entering the droplets by direct impingement of the Ga and As fluxes and (b) the Ga atoms entering the droplets by diffusion on the SiO_2_-terminated Si substrate (for short NWs) and on the NW facets (more details can be found in the ESI section†). Motivated by the droplet stability at a solid angle, an original feature of the model revealed by Vettori *et al.*^45^ is the existence of an upper-limit wetting angle for the droplet (defined as *β*_max_ in the previous sections). As a consequence, besides the classical axial NW growth the model is able to predict conditions that trigger both the axial and the lateral NW growth depending on droplet evolution. In our case, we expect the numerical simulations to confirm the unique (critical) value of the wetting angle that characterizes the ZB-to-WZ and WZ-to-ZB phase transitions. Fig. 7 shows the time-evolution for: (a) the Ga and As amounts of atoms feeding the droplets, (b) the droplet and NW radii, (c) the wetting angle of the droplets and (d) the NW length. The Ga flux closing between 240 and 300 seconds induces a decrease of the droplet radius from 25 nm to 18 nm (shown in (b)) and of the wetting angle from 134° to 98° (shown in (c)). Moreover, the decrease of the wetting angle (or droplet volume) induces a decrease of the droplet capture surface for As atoms and, as a consequence, a slightly lower axial growth rate (shown in (d)). When the Ga flux is opened at 300 seconds the droplet radius and the wetting angle increase again. At 370 seconds the maximum wetting angle *β*_max_ is attained (∼135°) so that the NW radius increases (shown in (b)) in order to accommodate a higher droplet volume.

The vertical dotted lines in Fig. 7(c) and (d) correspond to *t* = 257 seconds and *t* = 326 seconds when the ZB-to-WZ and WZ-to-ZB phase transitions occur as measured in Fig. 6. The corresponding ZB and WZ segment lengths predicted by the numerical simulation are 450 nm and 106 nm, respectively, in good agreement with the TEM measurements of 440 ± 70 nm and 120 ± 20 nm, respectively. The numerical method predicts values of the wetting angle equal to 126° for the ZB-to-WZ transition and to 124° for the WZ-to-ZB transition, in agreement with the results recently reported by Kim *et al.*^31^ and measured by *ex situ* TEM.^32^ It should also be noted that this critical wetting angle is close to the values reported for Au-catalyzed GaAs NWs.^29,30^

## 4. Conclusion

With the indexation of the ZB and WZ spots observed in the RHEED pattern during the growth of the self-catalyzed GaAs NWs, we have been able to follow the total consumption of the Ga droplets at the end of the growth when the Ga flux is stopped, as well as establishing a procedure capable of producing an extended WZ segment inside ZB GaAs NWs using only an *in situ* characterization technique such as the RHEED technique. All the results were based on the *in situ* RHEED pattern analysis and were validated and characterized using TEM measurements. It should be mentioned that the influence of the volume of the Ga droplets on the RHEED spot intensities due to shadowing effects could be investigated using the same method presented in this work. Finally, using numerical simulations for the NW growth and our *in situ* RHEED measurements, a critical wetting angle of the Ga droplets of about 125° was found for the ZB-to-WZ and WZ-to-ZB phase transitions.

## Conflicts of interest

There are no conflicts to declare.

## Supplementary Material

NA-002-D0NA00273A-s001

NA-002-D0NA00273A-s002

NA-002-D0NA00273A-s003

## References

[cit1] Gudiksen M. S., Lauhon L. J., Wang J., Smith D. C., Lieber C. M. (2002). Nature.

[cit2] Mårtensson T., Svensson C. P. T., Wacaser B. A., Larsson M. W., Seifert W., Deppert K., Gustafsson A., Wallenberg L. R., Samuelson L. (2004). Nano Lett..

[cit3] Arbiol J. and Xiong Q., Semiconductor Nanowires Materials, Synthesis, Characterization and Applications, Elsevier., 2015

[cit4] Dayeh S. , Fontcuberta I Morral A. and Jagadish C., Semiconductor Nanowires: Materials, Synthesis, Characterization and Applications, Academic Press., 2016

[cit5] Novel compound semiconductor nanowires: materials, devices, and applications, ed. F. Ishikawa and I. A. Buyanova, Pan Stanford Publishing, Singapore, 2018

[cit6] Wagner R. S., Ellis W. C. (1964). Appl. Phys. Lett..

[cit7] Glas F., Harmand J.-C., Patriarche G. (2007). Phys. Rev. Lett..

[cit8] Belabbes A., Panse C., Furthmüller J., Bechstedt F. (2012). Phys. Rev. B: Condens. Matter Mater. Phys..

[cit9] Capiod P., Xu T., Nys J. P., Berthe M., Patriarche G., Lymperakis L., Neugebauer J., Caroff P., Dunin-Borkowski R. E., Ebert Ph., Grandidier B. (2013). Appl. Phys. Lett..

[cit10] Spirkoska D., Arbiol J., Gustafsson A., Conesa-Boj S., Glas F., Zardo I., Heigoldt M., Gass M. H., Bleloch A. L., Estrade S., Kaniber M., Rossler J., Peiro F., Morante J. R., Abstreiter G., Samuelson L., Fontcuberta i Morral A. (2009). Phys. Rev. B: Condens. Matter Mater. Phys..

[cit11] Ahtapodov L., Todorovic J., Olk P., Mjåland T., Slåttnes P., Dheeraj D. L., van Helvoort A. T. J., Fimland B.-O., Weman H. (2012). Nano Lett..

[cit12] Vainorius N., Jacobsson D., Lehmann S., Gustafsson A., Dick K. A., Samuelson L., Pistol M.-E. (2014). Phys. Rev. B: Condens. Matter Mater. Phys..

[cit13] Mante P.-A., Lehmann S., Anttu N., Dick K. A., Yartsev A. (2016). Nano Lett..

[cit14] Zou X., Chen X., Huang H., Xu Y., Duan W. (2015). Nanoscale.

[cit15] Al-Zahrani H. Y. S., Pal J., Migliorato M. A., Tse G., Yu D. (2015). Nano Energy.

[cit16] Calahorra Y., Guan X., Halder N. N., Smith M., Cohen S., Ritter D., Penuelas J., Kar-Narayan S. (2017). Semicond. Sci. Technol..

[cit17] Knutsson J. V., Lehmann S., Hjort M., Lundgren E., Dick K. A., Timm R., Mikkelsen A. (2017). ACS Nano.

[cit18] Jabeen F., Grillo V., Rubini S., Martelli F. (2008). Nanotechnology.

[cit19] Cirlin G. E., Dubrovskii V. G., Samsonenko Yu. B., Bouravleuv A. D., Durose K., Proskuryakov Y. Y., Mendes B., Bowen L., Kaliteevski M. A., Abram R. A., Zeze D. (2010). Phys. Rev. B: Condens. Matter Mater. Phys..

[cit20] Plissard S., Dick K. A., Larrieu G., Godey S., Addad A., Wallart X., Caroff P. (2010). Nanotechnology.

[cit21] Rudolph D., Hertenberger S., Bolte S., Paosangthong W., Spirkoska D., Döblinger M., Bichler M., Finley J. J., Abstreiter G., Koblmüller G. (2011). Nano Lett..

[cit22] Yu X., Wang H., Lu J., Zhao J., Misuraca J., Xiong P., von Molnár S. (2012). Nano Lett..

[cit23] Heon Kim Y., Woo Park D., Jun Lee S. (2012). Appl. Phys. Lett..

[cit24] Scarpellini D., Fedorov A., Somaschini C., Frigeri C., Bollani M., Bietti S., Nöetzel R., Sanguinetti S. (2017). Nanotechnology.

[cit25] Dubrovskii V. G., Sibirev N. V., Harmand J. C., Glas F. (2008). Phys. Rev. B: Condens. Matter Mater. Phys..

[cit26] Krogstrup P., Curiotto S., Johnson E., Aagesen M., Nygård J., Chatain D. (2011). Phys. Rev. Lett..

[cit27] Dubrovskii V. G., Cirlin G. E., Sibirev N. V., Jabeen F., Harmand J. C., Werner P. (2011). Nano Lett..

[cit28] Krogstrup P., Jørgensen H. I., Johnson E., Madsen M. H., Sørensen C. B., i Morral A. F., Aagesen M., Nygård J., Glas F. (2013). J. Phys. D: Appl. Phys..

[cit29] Jacobsson D., Panciera F., Tersoff J., Reuter M. C., Lehmann S., Hofmann S., Dick K. A., Ross F. M. (2016). Nature.

[cit30] Harmand J.-C., Patriarche G., Glas F., Panciera F., Florea I., Maurice J.-L., Travers L., Ollivier Y. (2018). Phys. Rev. Lett..

[cit31] Kim W., Dubrovskii V. G., Vukajlovic-Plestina J., Tütüncüoglu G., Francaviglia L., Güniat L., Potts H., Friedl M., Leran J.-B., Fontcuberta i Morral A. (2018). Nano Lett..

[cit32] Panciera F., Baraissov Z., Patriarche G., Dubrovskii V. G., Glas F., Travers L., Mirsaidov U., Harmand J.-C. (2020). Nano Lett..

[cit33] Krogstrup P., Popovitz-Biro R., Johnson E., Madsen M. H., Nygård J., Shtrikman H. (2011). Nano Lett..

[cit34] Ambrosini S., Fanetti M., Grillo V., Franciosi A., Rubini S. (2011). AIP Adv..

[cit35] Krogstrup P., Hannibal Madsen M., Hu W., Kozu M., Nakata Y., Nygård J., Takahasi M., Feidenhans’l R. (2012). Appl. Phys. Lett..

[cit36] Rieger T., Lepsa M. I., Schäpers T., Grützmacher D. (2013). J. Cryst. Growth.

[cit37] Munshi A. M., Dheeraj D. L., Todorovic J., van Helvoort A. T. J., Weman H., Fimland B.-O. (2013). J. Cryst. Growth.

[cit38] Bastiman F., Küpers H., Somaschini C., Dubrovskii V. G., Geelhaar L. (2019). Phys. Rev. Mater..

[cit39] Jo J., Tchoe Y., Yi G.-C., Kim M. (2018). Sci. Rep..

[cit40] Fontcuberta i Morral A., Spirkoska D., Arbiol J., Heigoldt M., Morante J. R., Abstreiter G. (2008). Small.

[cit41] Küpers H., Bastiman F., Luna E., Somaschini C., Geelhaar L. (2017). J. Cryst. Growth.

[cit42] Fouquat L., Vettori M., Botella C., Benamrouche A., Penuelas J., Grenet G. (2019). J. Cryst. Growth.

[cit43] Dubrovskiĭ V. G., Sibirev N. V., Suris R. A., Cirlin G. É., Ustinov V. M., Tchernysheva M., Harmand J. C. (2006). Semiconductors.

[cit44] Tchernycheva M., Travers L., Patriarche G., Glas F., Harmand J.-C., Cirlin G. E., Dubrovskii V. G. (2007). J. Appl. Phys..

[cit45] Vettori M., Danescu A., Guan X., Regreny P., Penuelas J., Gendry M. (2019). Nanoscale Adv..

