# Supplementary material for: Crystal phase engineering of self-catalyzed GaAs nanowires using a RHEED diagram
Source: Nanoscale Adv. 2020 Apr 13;2(5):2127–34. doi: 10.1039/d0na00273a (PMC9418245; doi:10.1039/d0na00273a)
Supplement: NA-002-D0NA00273A-s003 [file NA-002-D0NA00273A-s003.pdf]

Electronic Supplementary Material (ESI) for Nanoscale Advances.
This journal is © The Royal Society of Chemistry 2020

Supplemental Information

1 Probed depth of the electron beam

The information obtained on the RHEED diagram is dependent on the probed depth of the electron beam. However, this probed depth can vary with the density, length of the NWs as well as the incidence angle of the electron beam. In our experiments, NWs are about 1.1 μm long with a diameter in the 40-60 nm range and a density of about 1 NW/μm².

The probed depth of the electron beam could be estimated using the intensity ratios (IR) curves (Figure 1 S.I. (a)) and the short ZB/WZ mixing phase and WZ segments which are grown in the early growth stages at the bottom of the NW. Indeed, the peak on the WZ IR (Figure S.I. (b)) will arise once the WZ segment ended and the ZB-to-WZ phase transition occurred. The WZ spot induced by this growth will remain (Figure 1 S.I. (c)) until the segment is no longer probed by the electron beam (Figure 1 S.I. (d)). This remaining time was estimated using the RHEED movie to 290 seconds which corresponds to about 500 nm, accordingly to the simulations on Figure 7 (d).

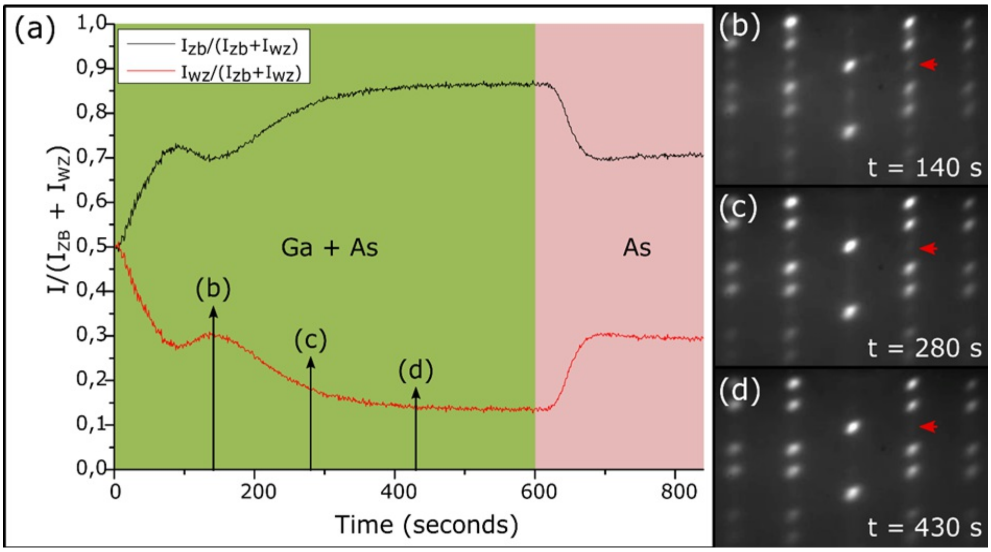

Figure 1. (a) $\frac{I_{ZB}}{I_{ZB}+I_{WZ}}$ and $\frac{I_{WZ}}{I_{ZB}+I_{WZ}}$ intensity ratios as a function of the growth time. In green the grown under Ga and As fluxes, in red the grown under As flux only. Black arrows the RHEED snapshots at: (b) $t = 140s$, (c) $t = 280s$ and (d) $t = 430s$. Red arrows point the (10-12) spot.

The movie recorded during the growth and used to plot the intensity ratio (IR) curves shown in Figure 4 (a). The film has been accelerated 50 times for convenient reasons.

Movie_x50_Fig4.avi

The movie recorded during the growth and used to plot the IR curves shown in Figure 5 (a). The film has been accelerated 50 times for convenient reasons.

Movie_x50_Fig5.avi

2 Model for NW growth

The results presented in Fig. 7 are obtained by the numerical implementation of a model for NW growth detailed in [1] but for completeness we recall here the main features of the model.

Assume given at time t : the NW length further denoted $L(t)$, the NW radius under the droplet denoted $r(t)$, the value for the critical concentration $c^\star$, the values of Ga and As nominal fluxes (i.e. number of atoms/(unit time)×(unit surface normal to the flux direction)) and the radius and the As concentration of the droplet further denoted $R(t)$ and $c(t)$ and such that $c(t) \leq c^\star$ (subcritical concentration). Obviously, the knowledge of the concentration in the droplet and its volume allow to compute the amount of Ga and As atoms in the droplet, further denoted $Q_{\mathrm{Ga}}(t)$ and $Q_{\mathrm{As}}(t)$.

The evolution of the NW is driven by the amount of Ga and As atoms feeding the droplet and the following two requirements: (a) solidification occurs only when the droplet concentration attains the critical value $c^\star$ and (b) the droplet wetting angle is such that $\beta_{\min} \leq \beta(t) \leq \beta_{\max}$ where the two limit angles $\beta_{\min}$ and $\beta_{\max}$ depend on the crystallographic properties of the NW.

The NW growth process is described as follows: in a small time-interval $(t, t+\delta t)$ we assume that the droplet incorporates an amount of Ga atoms, denoted $\Delta Q_{\mathrm{Ga}}(t)$ coming from one of the following sources: (i) direct impingement, (ii) diffusion along the the NW facets with diffusion length λ_{facet} and (iii) diffusion on the substrate, caracterized by a diffusion length $\lambda_{\mathrm{substrate}}$. Obviously, the last contribution is lost when the NW length overcome the diffusion length on the NW facets. Meanwhile, we assume that the amount of As atoms feeding the droplet, further denoted $\Delta Q_{\mathrm{As}}(t)$ is due to direct impingement only.

Two situations may occur:

1. The As "potential concentration" in the droplet at $t+\delta t$, defined as

$$\frac{Q_{\mathrm{As}}(t)+\Delta Q_{\mathrm{As}}(t)}{Q_{\mathrm{As}}(t)+\Delta Q_{\mathrm{As}}(t)+Q_{\mathrm{Ga}}(t)+\Delta Q_{\mathrm{Ga}}(t)}$$

   is $\leq c^\star$. In this case no solidification occurs and we distinguish two sub-cases: (i) If the droplet can be pinned on the top of the NW with an wetting angle $\leq \beta_{\max}$ then the droplet volume increases but the NW length remains constant (no axial growth). (ii) If the droplet volume is such that the wetting angle is greater than $\beta_{\max}$ the computation is stopped from droplet stability considerations.

2. The As "potential concentration" in the droplet is such that

$$\frac{Q_{\mathrm{As}}(t)+\Delta Q_{\mathrm{As}}(t)}{Q_{\mathrm{As}}(t)+\Delta Q_{\mathrm{As}}(t)+Q_{\mathrm{Ga}}(t)+\Delta Q_{\mathrm{Ga}}(t)} > c^\star.$$

   In this case there exist unique equal quantities Q of Ga and As atoms such that

$$\frac{Q_{\mathrm{As}}(t)+\Delta Q_{\mathrm{As}}(t)-Q}{Q_{\mathrm{As}}(t)+\Delta Q_{\mathrm{As}}(t)+Q_{\mathrm{Ga}}(t)+\Delta Q_{\mathrm{Ga}}(t)-2Q} = c^\star.$$

   Otherwise stated, only the excess of Ga and As atoms (due to supersaturation) will contribute to the solidification process. But the volume of solid material can contibute to both axial and radial growth, depending on the remaining volume of the droplet which now includes a total amount of Ga and As atoms given by $Q_{\mathrm{As}}(t)+\Delta Q_{\mathrm{As}}(t)+Q_{\mathrm{Ga}}(t)+\Delta Q_{\mathrm{Ga}}(t)-2Q$. If this volume can be pinned on the top of the NW with an angle $\beta(t+\delta t)$ such that $\beta_{\min} \leq \beta(t+\delta t) \leq \beta_{\max}$ then we have only axial growth. We update the NW length $L(t+\delta t)$ and the droplet radius $R(t)$. If not, two sub-cases may occur:

   a) If $\beta(t+\delta t) > \beta_{\max}$ we have both axial growth and inverse tappering. The inverse tappering is such that the NW radius $r(t+\delta t)$ is the unique value that can support the fixed droplet volume at wetting angle $\beta_{\max}$. As a consequence, the axial growth will place the solid material in a truncated cone geometry with lower radius $r(t)$, upper radius $r(t+\delta t)$ and height determined by the amount of equal solid quantities Q of Ga and As atoms.

b) If $\beta(t+\delta t) < \beta_{\text{min}}$ we have axial growth and direct tappering and the situation is similar to the previous one. There is an unique value of $r(t+\delta t)$ that can support the droplet at a wetting angle β_{min}. The solid material will be placed in a truncated cone geometry with lower radius $r(t)$, upper radius $r(t+\delta t) < r(t)$ and height determined by the amount of equal solid quantities Q of Ga and As atoms.

We also stop the algorithm in the unrealistic situations when the NW radius becomes too large or too small. Numerical results in Fig. 7 were obtained assuming $c^{\star} = 0.01$, $\beta_{\text{min}} = 50°$, $\beta_{\text{max}} = 140°$ under Ga and As fuxes calibrated to values that provide GaAs growth rate equal to 0.5 ML/s on the planar Si(111) substrate.

References

1. M. Vettori et al, Impact of the Ga flux incidence angle on the growth kinetics of self-assisted GaAs nanowires on Si(111), *Nanoscale Advances* (in press), **2019**.
